# Supplementary material for: Patterns of Intron Gain and Loss in Fungi
Source: PLoS Biol. 2004 Nov 30;2(12):e422. doi: 10.1371/journal.pbio.0020422 (PMC532390; doi:10.1371/journal.pbio.0020422)
Supplement: Table S1 — Also available at http://genes.mit.edu/NielsenEtAl/. (4.3 MB ZIP). [file pbio.0020422.st001.zip › NielsenEtAl/html/442.html]

AN3795.1.NCU02114.1.MG03595.1.FG00941.1


```
 CLUSTAL W (1.82) Multiple Sequence Alignments - Introns Inserted


Sequence 1: NCU02114.1	416 aa
Sequence 2: MG03595.1	406 aa
Sequence 3: FG00941.1	399 aa
Sequence 4: AN3795.1	419 aa
Alignment Length: 428 aa
Number Identitical Residues: 174 aa
Alignment Score (without introns) 8683


MG03595.1 	MAYHHHSRA----DGTFFVE-SDDIAMSRMSAREAAKILARNRREVIGNELSRLAGEEYL
NCU02114.1	MVTSRHN------DETFFVE-SDDQPAPRVNVRDAAKLIARNKQEMIANNLSRLACDEYL
FG00941.1 	MAYSQHD-------DTFFVE-SDE-PQHRITAQEAARMMARQRQDIIGVELSRLAGDEYL
AN3795.1  	MAYHKRPTSSYQPCDSYFVESYDDFTVPRMDPKEHAKLVARERQYAMADELSRVTSEEYR
          	*.  ::  :: ..  ::***: *: .  *:  :: *:::**:::  :. :***:: :** 

MG03595.1 	EDIMQHMRHME0DETLPDASLIDMQREIQWFMRPYLIDFLVEAHAAFQLLPETLFLTVNL
NCU02114.1	EDIMQHIRHME0DETLPDANLIDMQREIQWFMRPYLIDFLIEAHAAFSLLPETLFLTVNL
FG00941.1 	EDIMQHMRQME0DETLPDASLIDMQREIQWFMRPYLIDFLIEAHAAFGLLPETLFLTVNL
AN3795.1  	DDILSHMLDMD0ASTLPDVESIDIQTEIQWFMRPYLLDFLIEAHTAFQLLPSTLFLTVNL
          	:**:.*: .*:  .****.. **:* **********:***:***:** ***.********

MG03595.1 	LDRYCSKRVVYKQHYQLVGCAALLIAAKYGDKKDRVPQINELNNMCCGLYDAGMFTQMEM
NCU02114.1	LDRYCSKRVVYKQHYQLVGCAALLIAAKYGDKKDRVPQINELNNMCCGLYDAGMFTQMEM
FG00941.1 	LDRYCSKRVVYKQHYQLVGCAALLIAAKYGDKKDRVPQIHELNNMCCGLYDAGMFTQMEM
AN3795.1  	LDRYCSKRVVYKRHYQLVGCAALLIAAKYGDKKDRVPTIRELKSMCCSLYDDDMFIQMEW
          	************:************************ *.**:.***.*** .** *** 

MG03595.1 	HVLNTLDWTVGHPTVDFFAQLIVAEERDDKEVEYMAAYISEISLYYRDFVSTKPSTMARA
NCU02114.1	HVLNTLDWNIGHPTVDFFTQLIVAEERDSRDVEHMAAYICEIALYHRDFVSTKPSIMARV
FG00941.1 	HVLNTLDWIIGHPTVDFFSQLMVAEEGDDQEVSHMAAYLCEIALYHRDFVSTKPSVLARS
AN3795.1  	HVLQTLGWTIGHPTVDSFLQTAVMDTPYDPEVEHLALYIAEISLFHREFVSKPSSDLARA
          	***:**.* :****** * *  * :   . :*.::* *:.**:*::*:***. .* :** 

MG03595.1 	SLALARAILGRQEVNDGEWD-HDQNATLVTLSQHVHQPSVTLSRKYASTHYLQVSNKLSD
NCU02114.1	SLVLARAILGKPEVNDGEWD-QVENATLLALSQHLHQPSVTLSRKYSSAYLSKVSGKLAD
FG00941.1 	SLALARAILGRTEVNDGGWD-QTENVTLITLSHHLHQPSPTLARKYSTTGFSRVSQKLAE
AN3795.1  	SLALARCILNRPQPRHTEWASQYDSMTLVGLSQQLHQPSQVLARKYSSSHYSRVSKILEQ
          	**.***.**.: : ..  * :: :. **: **:::**** .*:***:::   :**  * :

MG03595.1 	FLAHQAAIN-RAAALPENPLTPTTNELMAAKANIYSTPQKGLGSVPGVADGYLTPPITPD
NCU02114.1	FLAQQAAINRRGGAPPSSPGELTPNSSKAS--NVYSTPHKGLGAVPGAADGYMTPPITPD
FG00941.1 	FMAEQAAIARRAAS--ANPATPLAEPINKHTSNIYSTPSKGHSAMG--FDGYLTPPITPD
AN3795.1  	FLARQASIASYTPP--SPPSDVVNSESKPYEGEIGLATPQKAPHPTNMPHGYITPPITPE
          	*:*.**:*     .    *     .      .::  :. :      .  .**:******:

MG03595.1 	GQYGAPN------PHGLPPRCPVTPTPQ---HQQ-----QQHQQQQRYYPNCYQPTQQQQ
NCU02114.1	AASFAQNG--TMMKEYPAPRCPVTPTPQPNSHSQSYPPAAQHQTSASSTSSSTKAPSYFE
FG00941.1 	GNTLMGNHNLAKESYVLPPRCPVTPTPP--HHAAVY---GQQQQQNQYVGYVNQHGMNQ-
AN3795.1  	NEAFVNGNAGYVKAVPVPSACSPSPTPS---PSVQYVTHNPYQQESNYVTQQQYLQPQMS
          	      .          .. *. :***          .    * .              .

MG03595.1 	TSNHGSYL-
NCU02114.1	TAPSSQVTY
FG00941.1 	---------
AN3795.1  	FTSGY----
          	 :
```
